# Association of *LIN28B* Gene Polymorphisms with Intrauterine Adhesions in Patients after Curettage Abortion

**DOI:** 10.3390/biomedicines12092044

**Published:** 2024-09-09

**Authors:** Danting Shen, Cong Li, Shuhua Liu, Anping Lin, Bin Liu

**Affiliations:** 1 Department of Gynecology, Shenzhen Baoan Women’s and Children’s Hospital, 56 Yulv Road, Shenzhen 510181, China; 13590123096@163.com (D.S.); 15941291398@163.com (C.L.); liushuhuaxf@163.com (S.L.); anpinglin2022@163.com (A.L.); 2 Maternal & Child Health Research Institute, Shenzhen Baoan Women’s and Children’s Hospital, 56 Yulv Road, Shenzhen 510181, China

**Keywords:** curettage abortion, intrauterine adhesion, *LIN28B*, genetic polymorphisms, occurrence

## Abstract

***Background/Objectives***: Intrauterine adhesion (IUA) is characterized by endometrial fibrocyte hyperplasia. The *LIN28B* gene is associated with many proliferative diseases. However, its association with IUA is entirely unknown. We hypothesized that *LIN28B* gene polymorphisms are responsible for IUA susceptibility after curettage abortion. ***Methods***: In this genetic association study, We genotyped two common polymorphisms (rs369065 C>T and rs314280 A>G) in 107 patients with IUA and 270 controls without IUA after curettage abortion from a Chinese population between July 2022 and May 2023 and analyzed their associations with IUA risk using multiple logistic regression models. ***Results***: The carriers of genotype rs314280 AA of the *LIN28B* gene showed an increased risk of IUA (AOR [adjusted odds ratio] = 2.12, 95% CI [confidence interval] = 1.151–3.903), compared to GG+GA genotypes. Further stratification analyses showed that the deleterious role of the rs314280 AA genotype was more evident in patients with fewer than four pregnancies (AOR = 2.740, 95% CI = 1.355–5.540), fewer than two births (AOR = 2.676, 95% CI = 1.300–5.509), and fibrous (AOR = 2.082, 95% CI = 1.084–3.997) and muscular adhesions (AOR = 3.887, 95% CI = 1.116–13.540). However, the rs369065 T>C polymorphism of the *LIN28B* gene was not significantly associated with the occurrence of IUA. ***Conclusions***: The rs314280 AA genotype of the *LIN28B* gene is associated with an increased risk of IUA in patients after curettage abortion, especially in those with fewer pregnancies or parities and higher disease severity. Our findings implicate a precise choice of clinical counseling and decision-making of IUA, thereby protecting female reproduction.

## 1. Introduction

Intrauterine adhesions (IUA) are partial or total adhesions of the uterine cavity caused by damage to the uterine basal layer that induces endometrial fibrosis [1]. Its prevalence has recently increased in young women. Intrauterine scarring, fibrotic adhesion, and partial or complete endometrial dysfunction of IUA [2] can cause menstrual disorders, infertility, repeated abortion, and other pregnancy complications [3,4]. It is important to prevent IUA because it is difficult to cure.

The etiology of IUA is still unclear. However, pathogenesis includes active fibrocyte proliferation and neural reflexes [3]: basal layer injury of the endometrium is primarily caused by pregnancy-related curettage [5] in addition to postoperative inflammation and/or infection [6], and damaged proliferative activity of local fibrocytes induces fibrinogen aggregation [3]. Therefore, fibrocyte proliferation is widely considered a critical factor in IUA after abortion. To prevent the incidence of IUA, biomarkers for the susceptible population are warranted.

*LIN28B*, a 27084 Da RNA-binding protein, is a key etiological factor in the occurrence and recurrence of many proliferative diseases by selectively inhibiting the biosynthesis of the let-7 family [7,8,9,10,11]. Its encoded gene (NCBI Gene: 389421) spans 14.6 kb on chromosome 6q16.3-21 and contains seven exons. LIN28B regulates endometrial cell proliferation [12] through the derepression of let-7 targets [13] and is activated in the early reprogramming of human fibroblasts [14] involved in the development of IUA.

We have reported that *LIN28B* polymorphisms are associated with the postoperative recurrence of endometrial polyps [13], implicating the gene’s role in the hyperplasia of endometrial cells and endometrial fibrocytes. However, no studies have reported an association between *LIN28B* polymorphisms and IUA. We hypothesized that genetic variants of the *LIN28B* gene can lead to IUA in patients who underwent curettage abortion.

Therefore, we genotyped rs369065 C>T and rs314280 A>G polymorphisms in 383 reproductive female patients who underwent curettage abortion in a southern Chinese population between 2022 and 2023 and analyzed their associations with the risk of postoperative occurrence of IUA. Our study is the first to report the associations between *LIN28B* gene polymorphisms and IUA.

## 2. Materials and Methods

### 2.1. Study Population

This genetic association study was conducted in accordance with the reporting guidelines and checklist of criteria set in STrengthening the REporting of Genetic Association Studies (STREGA, Supplementary Table S1).

This study was a nested case–control study from a prospective clinical cohort. Because curettage abortion is a major risk factor for IUA [15], and there are about 13~14 k patients who undergo curettage abortion annually at the Department of Gynecology in the study Hospital, we selected females who had undergone curettage abortion as the source of the study population. Curettage by vacuum aspiration, the predominant abortion technique in the first trimester, was performed up to 10 gestational weeks in these patients. After intravenous or cervical anesthesia and dilation of the cervix, a tube (vacurette) is inserted into the uterus, which is connected to a suction device attached to a collection bottle. The procedure was performed by 1~2 professional and experienced gynecologists. Under ultrasound monitoring, the gynecologist gently scratched the entire uterine cavity 2–3 times until the uterine wall had a rough feeling. Among these patients, those with hypomenorrhea or amenorrhea after curettage abortion and/or suspected IUA by routine ultrasound will undergo a hysteroscopy to diagnose IUA. We excluded patients with a history of chronic pelvic inflammation, existing IUA prior to curettage abortion, and intrauterine operation after curettage abortion. According to the Practice Guidelines on IUA developed in collaboration with AAGL (American Association of Gynecologic Laparoscopists) and ESGE (the European Society of Gynaecological Endoscopy) [15], there are various classification systems, making comparisons between studies hard to interpret. However, the key features of these systems include the type and area of adhesions, which were used to classify IUA in this study. We recruited all 113 eligible IUA patients aged 21~44 years who underwent office hysteroscopy with cold blade scissoring between July 2022 and May 2023. With formal consent, the patients’ information, such as age, menstrual pattern, parity, gravidity, and history of gynecological diseases, was collected from their electronic medical records. In addition, 5 mL of heparin anticoagulant blood was collected to perform blood analyses and genotyping.

During the same time, 270 patients aged 17~44 years without IUA after curettage abortion were recruited as the control group, who were followed until February 2024 with a median 10-month follow-up, to exclude those with symptoms such as decreased menstruation, amenorrhea, and suspected IUA by transvaginal three-dimensional ultrasound after curettage abortion. The control group was age-frequency matched (more or less than 30 years old) with the case group. Their medical information and blood samples were also collected before curettage abortion after signing consent statements. Among these controls, the informed consents for abortion and investigation in three subjects aged 17 were signed by their parents.

The Ethics Committee of the study hospital approved this study (IRB No: LLSC-2022-01-04-10-KS).

### 2.2. Single-Nucleotide Polymorphism Selection and Genotyping

This study selected rs314280 G>A and rs369065 T > C in the LIN28B gene as described in our previous study [13], mainly based on the MAF (minor allele frequencies > 0.2) and linkage disequilibrium (*R*^2^ < 0.8) in Chinese.

DNA extraction and SNP (single-nucleotide polymorphism) genotyping were conducted as described previously [16] using the MagaBio plus Blood DNA Kit (Bioer Technology, Hangzhou, China) and Agenda Massarray platform (CapitalBio Technology, Beijing, China). The results of repeated genotyping on a random ~20% (76/383) of the samples were 100% concordant. All genotyping data of rs314280 G>A and rs369065 T>C polymorphisms were used for further analyses.

### 2.3. Statistical Analysis

Two different researchers double-checked the data used for analyses from the original medical records to avoid bias. The variables in the present study are all important clinical indices of IUA. There were no missing data in this study because the ineligible IUA and control cases were excluded. The Chi-Square test, Adjusted, and Fisher’s exact test were used to compare the differences between the IUA and control group regarding demographic characteristics. The Hardy–Weinberg equilibrium (HWE) tests of both polymorphisms applied the Chi-Square goodness of fit test in the controls. The IUA risk of different genotype groups were analyzed by the Breslow-Day test, multiplicative interaction, and multiple logistic regression analysis by adjusting for age. Further stratification analyses by age, gravidities, parities, extent of cavity, and type of adhesions were performed as well. We also performed the false positive report probability (FPRP) test. All tests have two-sided α = 0.05 as the statistical significance level using SPSS 18.0 software (IBM, Armonk, NY, USA).

## 3. Results

### 3.1. Characteristics of the Study Population

As shown in Table 1, patients with endometritis, pelvic infection, and parities less than two times were more likely to have the occurrence of IUA (*p* values were 0.046, <0.001, and <0.001, respectively). Additionally, the menstrual volumes of patients with IUA decreased significantly (*p* values < 0.001). However, the differences in other variables distributions between the IUA and non-IUA patients were not statistically significant (all *p* values > 0.05).

### 3.2. Association of LIN28B Polymorphisms with the Risk of IUA after Abortion

We analyzed the association of two common polymorphisms of the *LIN28B* gene (rs314280 G>A and rs369065 T>C) with the risk of uterine adhesion in a multivariate logistic regression model among the IUA and non-IUA patients (Table 2). The genotype frequencies of the study polymorphisms were both in accordance with HWE in the control group (*p* values were 0.245 and 0.410, respectively). To control statistical bias, we adjusted confounding factors in the multiple logistic model using age. As shown in Table 2, we found that the risk of IUA with the rs314280 AA genotype significantly increased (adjusted odds ratio [OR] = 2.255, 95% confidence interval [CI] = 1.178–4.319) compared with the rs314280 GG genotype. Similar results were found in the additive genetic model (GG vs. GA vs. AA, adjusted OR = 1.413, 95% CI = 1.036–1.928). However, the dominant genetic model did not show a significant association (GA+AA vs. GG, adjusted OR = 1.326, 95% CI = 0.803–2.187). In the recessive genetic model, the harmful genotype rs314280 AA had a significantly increased risk of uterine adhesion (adjusted OR = 2.12, 95% CI = 1.151–3.903) compared to other genotypes. According to the above analysis, the risk of IUA was particularly evident in patients with the genotype rs314280 AA. However, there was no significant association between the rs369065 T>C polymorphism and the occurrence of IUA (*p* values > 0.05 in all genetic models).

### 3.3. Stratification Analysis of LIN28B rs314280 Genotype and Risk of IUA

We further performed a stratification analysis of the associations between *LIN28B* rs314280 AA and IUA risk by age, gravidities, parities, extent of cavity involved, and type of adhesions. As shown in Table 3, the increased risks of IUA associated with the rs314280 AA genotype did not differ by age and extent of cavity involved. However, the harmful role of genotype LIN28B rs314280AA for the risk of IUA was more significant in patients with fewer than four previous pregnancies (adjusted OR = 2.740, 95% CI = 1.355–5.540) and two births (adjusted OR = 2.676, 95% CI = 1.300–5.509), and fibrous (firm and dense) (adjusted OR = 2.082, 95% CI = 1.084–3.997) and muscular (dense) adhesions (adjusted OR = 3.887, 95% CI = 1.116–13.540). By Breslow-Day homogeneity and the multiplicative interaction test, age, gravity, and parity were not confounding factors affecting IUA. However, these results may be caused by the limited population in each subgroup.

## 4. Discussion

In this study, we found that the rs31428 AA genotype of the *LIN28B* gene was associated with an increased risk of IUA in reproductive-age patients after induced abortion. Stratified analyses showed that this harmful role of the rs31428 AA genotype was more pronounced in patients with fewer pregnancies and births, indicating genetic susceptibility in these patients. Therefore, patients with this harmful genotype should pay attention to the occurrence of IUA after curettage abortion, even if they have no prior pregnancy history. In addition, it has been found that the higher the density of the adhesion type, the more obvious the correlation between *LIN28B* rs314280 G>A polymorphism and IUA, indicating the reliability of our findings.

To our knowledge, our study is the first to report the association between *LIN28B* gene polymorphisms and IUA risk. Fibrocyte proliferation is primarily responsible for the pathogenesis of IUA, which is characterized by the replacement of endometrial cells and stroma by non-vascular fibrous tissue and fusiform myofibroblasts [3]. The TGF-β1 pathway is considered the most important mechanism involved in endometrial fibrosis [17,18], while the LIN28B/let-7 axis can act on multiple steps of the TGF-β1 pathway, thus activating tissue fibrosis [14,19] and the pathogenesis of IUA. Additionally, the expression of LIN28B itself is regulated by TGF [19], which then affects let-7 family-mediated cell proliferation and differentiation through a reciprocal negative regulatory mechanism [20]. As analyzed by SNPinfo (https://manticore.niehs.nih.gov/ accessed on 1 September 2023), a new binding site of the transcriptional regulator AIRE is produced by the substitution G>A of rs314280. AIRE is a pivotal regulator of immunity and inflammation [21], suggesting that this SNP may be functionally related to IUA: a damaged endometrium can produce large amounts of AIRE after curettage abortion, which stimulates *LIN28B* gene expression by binding the new element introduced by the variant allele A, and then promotes the occurrence of IUA. A few studies have suggested that rs314280 polymorphism may affect susceptibility to radiation-induced pneumonitis [22] and chronic HBV infection [23]. On the contrary, there was not a significant association between the rs369065 T>C polymorphism and IUA risk in this study. This SNP is not linked to functional elements in the SNPinfo database. In our previous article [13], we speculated that its association with other proliferative diseases may be attributed to some nearby functional SNPs, such as rs221634. Therefore, the effect of the rs369065 T>C polymorphism is small and indirect, which can be indistinguishable in some studies with a limited sample. Based on the abovementioned reports, our data on the association between *LIN28B* rs314280 polymorphism and the risk of IUA are biologically plausible.

In the subgroup analysis, we found that the deleterious role of the rs314280 AA genotype was more evident in patients with filmy type adhesions and fewer previous pregnancies and births. First, the rs314280 AA genotype appears to be more harmful in patients with firm and dense and dense types of IUA, indicating a strong association between this SNP and disease severity. Second, more curettage abortions are a major risk factor of IUA. The association between the rs314280 AA genotype and IUA risk is more significant in patients with fewer previous pregnancies or births, implying genetic susceptibility in these patients. Therefore, this SNP should be a reliable marker of IUA risk. Our findings are important to protect female reproduction.

This study also had some limitations: this hospital-based genetic association study was restricted to a small Chinese population. However, the genotype frequencies of rs314280 and rs369065 among our subjects fit HWE well, and the A allele frequency of rs314280 in the controls of the present study (29.8%) is similar to those reported in a Chinese population (28.4% [23]) and in the CHB HAPMAP database (28.2%), suggesting that the subject selection is random. Second, there are many other ungenotyped polymorphisms that may be relevant and remain a risk for IUA. However, using this small sample, the study power achieved 97% to detect an OR of 2.120 for the rs314280 AA genotype (which occurred at a frequency of 10.4% in the non-IUA subjects) in this study. Therefore, it appears that our finding that the rs314280 AA genotype is associated with an increased risk of IUA is unlikely to have been by chance. Additionally, the FPRP analysis suggested by Wacholder et al. [24] showed that the FPRP value (0.10) for the abovementioned association was lower than the pre-set level of 0.20, suggesting the noteworthy of our findings. Therefore, further studies with a larger population and more SNPs are warranted.

## 5. Conclusions

We found that *LIN28B* polymorphisms were associated with IUA risk in reproductive-age women for the first time. Especially, the harmful rs314280 AA genotype was more evident in those with a more severe extent of IUA and those with fewer previous pregnancies or births. Our data suggest more precise alternatives to clinical counseling and decision-making for patients: the rs314280 AA genotype can be used as a biomarker for the population that is susceptible to IUA, who need careful choice of curettage abortion or need early prevention right after the first abortion, such as alginate carboxymethylcellulose hyaluronic acid. Multicenter studies with large-scale samples from different ethnic populations are warranted to confirm the findings.

## Figures and Tables

**Table 1:** Clinical and demographic characteristics of Intrauterine adhesion patients and control group.

Variables	Control *N* = 270 (*n*, %)	IUA *N* = 113 (*n*, %)	*p*
Age, years			
<30	83 (30.7)	28 (24.8)	
≥30	187 (69.3)	85 (75.2)	0.241 ^1^
Menstrual pattern			
Normal	264 (97.8)	33 (29.2)	
Hypomenorrhea or Amenorrhea	6 (2.2)	80 (70.8)	<0.001 ^1^
Gravidities			
<4	184 (68.1)	80 (70.8)	
≥4	86 (31.9)	33 (29.2)	0.261 ^1^
Parities			
<2	169 (62.6)	93 (82.3)	
≥2	101 (37.4)	20 (17.7)	<0.001 ^1^
With Endometriosis			
No	268 (99.3)	110 (97.3)	
Yes	2 (0.7)	3 (2.7)	0.312 ^2^
With adenomyosis			
No	270 (100.0)	112 (99.1)	
Yes	0 (0.0)	1 (0.3)	0.295 ^3^
With Pelvic infection			
No	269 (99.6)	109 (96.5)	
Yes	1 (0.4)	4 (3.5)	0.046 ^2^
With Endometritis			
No	270 (100.0)	103 (91.2)	
Yes	0 (0.0)	10 (8.8)	<0.001 ^2^
Extent of Cavity Involved			
1	70 (61.9)	-	-
≥2	43 (38.1)	-	-
Type of Adhesions			
Firm	4 (3.5)	-	-
Firm & Dense	96 (85.0)	-	-
Dense	13 (11.5)	-	-

^1^ Chi-squared test; ^2^ Adjusted Chi-squared test; ^3^ Fisher’s exact test. IUA, Intrauterine adhesions.

**Table 2:** Associations between *LIN28B* gene polymorphisms and occurrence of IUA.

	Genotypes	Control (*n* = 270)	IUA (*n* = 113)	OR (95% CI)	AOR (95% CI) ^2^
rs314280 G>A (HWE ^1^ = 0.245)					
	GG	137 (50.7)	48 (48.3)	1.000 (ref.)	1.000 (ref.)
	GA	105 (38.9)	43 (38.6)	1.169 (0.721–1.896)	1.147 (0.706–1.864)
Co-dominant	AA	28 (10.4)	22 (19.5)	2.243 (1.17–34.287)	2.255 (1.178–4.319)
Additive	AA vs. GA vs. GG			1.416 (1.039–1.930)	1.413 (1.036–1.928)
Dominant	GA+AA vs. GG	133 (49.3)	65 (57.5)	1.395 (0.896–2.172)	1.326 (0.803–2.187)
Recessive	AA vs. GG+GA	28 (10.4)	22 (19.5)	2.089(1.137–3.839)	2.120 (1.151–3.903)
rs369065 T>C (HWE ^1^ = 0.410)					
	TT	97 (35.9)	36 (31.9)	1.000 (ref.)	1.000 (ref.)
	TC	135 (50.0)	59 (52.2)	1.178 (0.722–1.922)	1.178 (0.721–1.925)
Co-dominant	CC	38 (14.1)	18 (15.9)	1.276 (0.647–2.516)	1.268 (0.642–2.503)
Additive	CC vs. TC vs. TT			1.139 (0.823–1.577)	1.136 (0.820–1.574)
Dominant	TC+CC vs. TT	173 (64.1)	77 (68.1)	1.199 (0.752–1.914)	1.198 (0.750–1.914)
Recessive	CC vs. TT+TC	38 (14.1)	18 (15.9)	1.157 (0.629–2.128)	1.149 (0.624–2.116)

^1^ The observed genotype frequency in the study population was in accordance with the Hardy–Weinberg equilibrium (p^2^ + 2pq + p^2^ = 1) (*p* = 0. 245 for rs314280; *p* =0.410 for rs369065). ^2^ Adjusted age. IUA, Intrauterine adhesions. OR: Odds ratio. AOR, Adjusted odds ratio. HWE, Hardy–Weinberg equilibrium. Ref., reference.

**Table 3:** Stratification analysis of *LIN28B* rs314280 polymorphism by selected variables in IUA and non-IUA patients (*n*, %).

Variables	Control Group (*n* = 270)			IUA Group(*n* = 113)			AA vs. GG+GA	*p*_homo_ ^2^	*p*_inter_ ^3^
	GG	GA	AA	GG	GA	AA	AOR (95% CI) ^1^		
**Age, Years**									
<30	46 (55.4)	28 (33.7)	9 (10.8)	12 (42.9)	9 (32.1)	7 (20.5)	2.741 (0.912–8.235)		
≥30	91 (48.7)	77 (41.2)	19 (10.2)	36 (42.4)	34 (40.0)	15 (17.6)	1.895 (0.911–3.940)	0.583	0.584
Gravidities									
<4	95 (51.6)	70 (38.0)	19 (10.3)	34 (42.5)	27 (33.8)	19 (23.8)	2.740 (1.355–5.540)		
≥4	42 (48.8)	35 (40.7)	9 (10.5)	14 (42.4)	16 (48.5)	3 (9.1)	0.855 (0.216–3.381)	0.135	0.143
Parities									
<2	84 (49.7)	69 (40.8)	16 (9.5)	37 (39.8)	36 (38.7)	20 (21.5)	2.676 (1.300–5.509)		
≥2	53 (52.5)	36 (35.6)	12 (11.9)	11 (55.0)	7 (35.0)	2 (10.0)	0.900 (0.181–4.466)	0.181	0.191
Extent of Cavity Involved									
1	137 (50.7)	105 (38.9)	28 (10.4)	31 (44.3)	26 (37.1)	13 (18.6)	2.004 (0.975–4.120)	-	-
≥2	137 (50.7)	105 (38.9)	28 (10.4)	17 (39.5)	17 (39.5)	9 (20.9)	2.271 (0.985–5.238)	-	-
Type of Adhesions									
Firm	137 (50.7)	105 (38.9)	28 (10.4)	1 (25.0)	3 (75.0)	0 (0.0)	-	-	-
Firm & Dense	137 (50.7)	105 (38.9)	28 (10.4)	43 (44.8)	35 (36.5)	18 (18.8)	2.082 (1.084–3.997) ^1^	-	-
Dense	137 (50.7)	105 (38.9)	28 (10.4)	4 (30.8)	5 (38.5)	4 (30.8)	3.887 (1.116–13.540) ^1^	-	-

^1^ Adjusted age. ^2^ *p* value for Breslow-Day Homogeneity Test. ^3^ *p* value for Multiplicative interaction. IUA, Intrauterine adhesions. AOR, Adjusted odds ratio.

## Data Availability

The datasets used and/or analyzed during the current study are available from the corresponding author upon reasonable request.

## Supplementary Materials

The following supporting information can be downloaded at: https://www.mdpi.com/article/10.3390/biomedicines12092044/s1; Table S1: STROBE-STREGA checklist of this study.
